# Does an NKT-cell-based immunotherapeutic approach have a future in multiple myeloma?

**DOI:** 10.18632/oncotarget.7440

**Published:** 2016-02-17

**Authors:** Mérédis Favreau, Karin Vanderkerken, Dirk Elewaut, Koen Venken, Eline Menu

**Affiliations:** ^1^ Department of Hematology and Immunology, Myeloma Center Brussels, Vrije Universiteit Brussel (VUB), Brussels, Belgium; ^2^ Laboratory for Molecular Immunology and Inflammation, Department of Rheumatology, Faculty of Medicine and Health Sciences, VIB Inflammation Research Center and Ghent University, Ghent, Belgium

**Keywords:** myeloma, NKT cells, tumor immunity

## Abstract

Natural killer T (NKT) cells constitute a unique subset of innate-like T lymphocytes which differ from conventional T cells by recognizing lipid antigens presented by the non-polymorphic major histocompatibility complex (MHC) I-like molecule CD1d. Despite being a relatively infrequent population of lymphocytes, NKT cells can respond rapidly upon activation with glycosphingolipids by production of cytokines which aim to polarize different axes of the immune system. Due to their dual effector capacities, NKT cells can play a vital role in cancer immunity, infection, inflammation and autoimmune diseases. It is believed that modulation of their activity towards immune activation can be a useful tool in anti-tumor immunotherapeutic strategies. Here we summarize the characteristics of NKT cells and discuss their involvement in immunosurveillance. Furthermore, an update is given about their role and the progress that has been made in the field of multiple myeloma (MM). Finally, some challenges are discussed that are currently hampering further progress.

## NATURAL KILLER T CELLS: SUBSETS AND FUNCTION

Natural killer T (NKT) cells constitute a highly conserved heterogeneous subset of innate-like T lymphocytes. This small population owns unique phenotypic and functional properties that set them apart from conventional T cells by exhibiting characteristics of both the innate and the adaptive immune system [1, 2]. They diverge from conventional T cells by recognizing foreign and self (glyco)sphingolipid antigens presented by the non-polymorphic major histocompatibility complex (MHC) I-like molecule CD1d, expressed on professional antigen-presenting cells (APCs) [2, 3]. Originally NKT cells were defined as expressing both the CD3 and αβ T-cell receptors (TCR) and lineage markers from natural killer (NK) cells, such as CD56 or CD161 (human) and NK1.1 (murine). It is now generally accepted that this description is no longer accurate since these cells only seem to be a part of the broader NKT-cell family [4]. Moreover, NKT cells have a remarkable capacity to produce extensive amounts of cytokines upon stimulation to activate NK cells, dendritic cells (DC), regulatory and conventional T cells and B cells [5, 6]. Thereby enhancing a cascade of complementary cytokines and chemokines and stimulating additional populations to mediate immune surveillance [6]. Due to their broad cytokine profile, NKT cells can both exert an immune enhancing and immunosuppressive role and play therefore a vital role in various pathologies, such as cancer, infection, inflammation and autoimmune diseases [6-11]. Modulating their activity towards immune activation could be a useful tool for improving vaccines in cancer, infectious diseases and other therapeutic settings.

### Type I natural killer T cells

**Figure 1:**
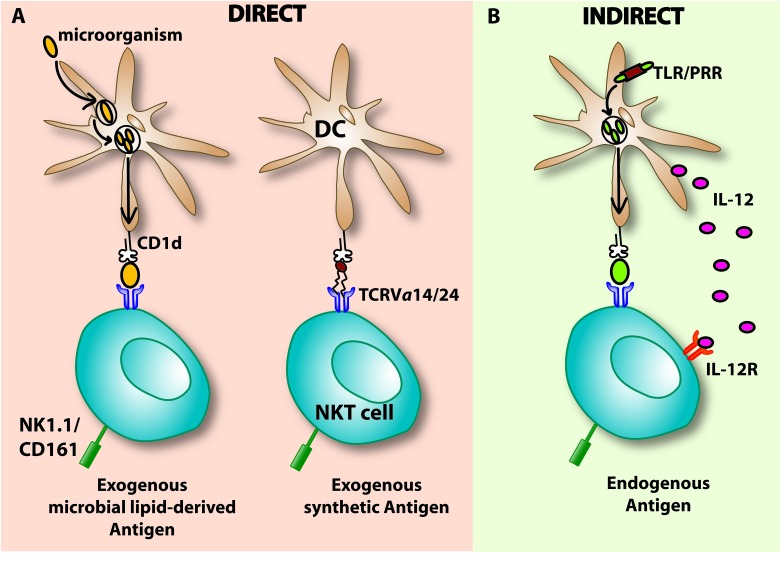
Major (I NKT) cell activation pathways **A.** Direct activation of iNKT cells occurs when the TCRVα14/24 interacts with a ligand presented by a CD1d molecule present on DCs or other APCs. DCs present exogenous glycosphingolipids such as the synthetic α-GalCer or microbial lipid-derived antigens and subsequently activate the iNKT cell. This CD1d dependent activation is followed by the secretion of cytokines such as IFN-γ and IL-4. **B.** Indirect activation of iNKT cells can be induced by cytokine secretion of DCs such as IL-12. Engagement of a microbial Ag to the pattern-recognition receptor (PRR) of the toll-like receptor (TLR) present on APCs (e.g DCs) triggers IL-12 co-stimulation. IL-12 secreted by DCs binds to its receptor, IL-12R, on iNKT cells which activates iNKT cells by inducing IFN-γ secretion. The activation occurs in the presence or absence of self or low affinity endogenous lipid antigens. Besides IL-12, also other cytokines such as IL-18 and type I-IFN (α & β) can be secreted and activate iNKT cells in a CD1d independent manner. iNKT, Invariant natural killer T; DCs, Dendritic cells; TCR, T cell receptor; α-GalCer, Alpha-Galactosylceramide; IL-12, Interleukin 12, IL-12R; Interleukin 12 receptor; TLR, Toll-like receptor; PRR, Pattern-recognition receptor; IFN, Interferon.

The type I NKT cells also referred to as “invariant” NKT cells (iNKT) are the main studied subpopulation of NKT cells and are usually linked to promotion of tumor immunity. They express a semi-invariant TCRα chain (Vα14-Jα18 in mice, Vα24-Jα18 in humans) paired with a heterogeneous Vβ chain repertoire (Vβ2, 7 or 8.2 in mice and Vβ11 in humans) [2]. Type I NKT cells often express other NK surface markers such as NK1.1 (in some mouse strains) or CD161 (in human), NKG2D, CD44, CD56, CD69, CD94, CD122 and members of the Ly49 family [8, 9]. iNKT cells can be further subdivided according to their CD4/CD8 co-receptor expression: CD4+ and CD4-CD8- (DN) subsets, and a small subset of CD8+ cells (only human) have been described [12-14]. Type I NKT cells are present in different tissues, such as the spleen (0,2-0,5% of the T lymphocytes), bone marrow, thymus, lymph nodes and blood (0,01-0,5% of the T lymphocytes) in mice [12, 15]. The highest frequency is found in the liver with around 10 to 30% of all T lymphocytes [16]. These hepatic iNKT cells possess a strong anti-tumor capacity and show different functional characteristics than the NKT cells from other tissues [15]. In humans, type I NKT cells appear to be approximately 10 times less abundant in the liver than in mice while for the spleen, bone marrow, blood and lymph nodes the ranges remain similar. The highest prevalence is found in the omentum, representing 10% of the white adipose tissue T cell population, whereas their frequency and number in the peripheral circulation vary widely between individuals [1, 8, 12, 17]. Identifying lipid antigens recognized by NKT cells is still an ongoing challenge. Type I NKT respond to α- and β-linked glycosphingolipids among which α-Galactosylceramide (αGalCer, KRN700), an exogenous synthetic glycolipid originally extracted from the marine sponge Agelas mauritianus or microorganisms symbiotic with the sponge [4, 18]. αGalCer is the most-well characterized agonist for type I NKT cells in humans and mice and shows a very potent capacity to induce cytokine release by iNKT cells [2]. Today, several new analogues of αGalCer, showing weaker or stronger agonistic potential, have been synthetized including, α-C-GalCer, naphthylurea 6”-derived α-GalCer (NU- α-GalCer), C20:2, DB06-1 and OCH [19]. Also microbial and self-glycolipid iNKT cell antigens such as ceramide structures (Sphingomonas species), diacylglycerols (Borrelia burgdorferi and Streptococcus pneumoniae), cholesteryl-sugars (Helicobacter pylori) but also phospholipids (Mycobacterium tuberculosis), the lysosomal glycosphingolipid isoglobotrihexosylceramide (iGb3) and the peroxisomal derived lipid plasmalogen lysophosphatidylcholine (lyso-PC) have been identified [20-26]. More recently, in their quest to find new endogenous ligands Kain et al. revealed the presence of mammalian α-linked glucosylceramides [27]. This defies the previous hypothesis where it was thought that humans were not able to make α-linked sugar moieties due to the presence of natural anti-α-linked sugar antibodies [28]. Direct CD1d-agonist stimulation of the TCR complex is accompanied by the rapid and robust release of T-helper 1 (Th1), Th2 and Th17 cytokines, including interferon-gamma (IFN-γ), interleukins (IL)-2, -4, -10, -13, -17, -21 and 22, granulocyte-macrophage colony-stimulating factor (GM-CSF) and tumor necrosis factor-alpha (TNFα) [3, 8, 11] (Figure 1). In addition, it was reported that cytolysis in a perforin-dependent manner, through the Fas-FasL axis or through expression of intracellular granzyme B is also promoted upon iNKT stimulation [29-31]. Similar to conventional T cells, through the engagement of costimulatory pathways such as CD40-CD40L and B7-CD28, DC are induced to maturate and secrete IL-12. In turn, IL-12 stimulates NK, NKT, and other T cells to produce IFN-γ which subsequently activates bystander cell activity and stimulates more downstream effector populations such as NK cells, CD8^+^ T cells and γδ cells [8]. Type I NKT cells are indirectly activated in response to pattern-recognition receptor (PRR) of toll-like receptor (TLR) signalling by APCs together with presentation of self-antigens through CD1d, inducing cytokine secretion by APCs such as IL-12, IL-18 and type I α/β IFNs [8, 22, 32, 33] (Figure 1). Also during inflammation, type I NKT cells can be stimulated in a TCR independent manner by different stimulatory and co-stimulatory signals, such as engagement of peroxisome proliferator activated receptor (PPAR)γ through bacterial products such as lipopolysaccharide (LPS) [32, 34]. Activation of Fcγ receptors by antigen-IgG complexes, interaction of NK1.1 receptors with their ligands on APCs and activation of TLRs of previously activated type I NKT cells also take part in additional activation mechanisms of type I NKT cells [32].

### Type II natural killer T cells

Type II NKT cells are a CD1d-restricted subset that expresses more diverse αβ-TCRs (for example Vα3.2Jα9 or Vα8 with Vβ8 TCRβ-chain). They have an activated or memory phenotype and many also express NK surface markers [3, 35]. Moreover, they have the ability to suppress autoimmunity and inhibit tumor rejection. In contrast to type I NKT cells, their distribution and physiological role is less understood. They compromise a minor subset in mice but constitute a major subgroup of the T cells in the bone marrow, liver and gut of humans [16, 36]. Type II NKT cells are non-reactive to α-GalCer. Currently, the most widely studied type II NKT antigen is sulfatide, a glycolipid abundantly present in neuronal tissue, liver, kidney and pancreas [37, 38]. Recent research also identified a range of hydrophobic antigens, such as lysosulfatide, lyso-PC, small aromatic (non-lipid) molecules and other lipids such as β-Glucosylceramide (β-GlcCer)(C24:0) and β-Galactosylceramide (β-GalCer) as being potential activators of type II NKT cells [39-42].

### Other invariant like T cells

Next to the different subsets of NKT cells, it is worth to briefly mention another population of semi-invariant T cells, called mucosal associated invariant T cells or MAIT cells. They are restricted to a monomorphic MHC I-like molecule MR1. Similar to NKT cells, they express an invariant TCRα-chain (Vα33Jα19 in mice and Vα7.2α19 in humans) combined with a limited but not invariant range of TCRβ-chains [43]. Rare in laboratory mice, they appear to be a very significant subset of T cells in humans, accounting for 1-10% of T cells in peripheral blood and being predominantly present in liver and mucosal tissues. Surprisingly, a completely new and unexpected class of antigens was shown to be presented by MRI molecules to MAIT cells, namely vitamin B2 (riboflavin) metabolites [44-46]. Although these cells are not CD1d restricted, their similarities with NKT cells are intriguing [47]. Research on MAIT cells has till now been hampered due to the lack of identification tools and the unknown nature of the antigens. However, very recently Reantragoon et al. were able to develop MR1 tetramers which allowed them to better phenotypically characterize human and mouse MAIT cells [48]. This development will lead to an increased understanding of the nature of MAIT cells. Also γδ T cells belong to this non-conventional invariant T cell group. Being innate-like lymphocytes, they differ from conventional αβ T cells since they do not express the CD4 and CD8 co-receptors but express Toll-like receptors and share a number of markers with NK cells [49]. Subsequently, antigen recognition by γδ TCR is not restricted to MHC molecules. γδ TCR recognize a diverse array of self and nonself-antigens, such as small peptides, soluble or membrane proteins, phospholipids, prenyl pyrophosphates, and sulfatides, while αβTCR bind peptides presented by MHC class I or class II molecules. In humans, γδ T cells represent 0.5-16% (on average: 4%) of all CD3+ cells in adult peripheral blood, and organized lymphoid tissues (thymus, tonsil, lymph nodes, and spleen), <5% in tongue and reproductive tract and 10-30% in intestine. In adult mice, 1-4% of all T cells in thymus, secondary lymphoid organs and lung are γδ T cells. γδ T cells are more abundant in other mucosal sites where they constitute 10-20% of all T cells in female reproductive organs, 20-40% of the intestinal intra-epithelial T cells and 50-70% of skin dermal T cells [50]. Moreover, the γδ TCR repertoire is restricted and depends on the tissue type and their localization. The conditions that lead to responses of γδ T cells are not fully understood, and current concepts of them are ‘first line of defense’, ‘regulatory cells’, or ‘bridge between innate and adaptive responses’.

## NATURAL KILLER T CELLS: IMPLICATIONS IN TUMOR IMMUNITY

### Enhancement of tumor immunity

Type I NKT cells are able to kill cancer cells directly or indirectly *via* the downstream activation of other innate and adaptive immune cells. Direct NKT lysis can be induced by perforin, *via* the Fas-FasL axis or through expression of intracellular granzyme B [29, 51]. *In vitro* observations demonstrated that tumor cells expressing CD1d were more prone to lysis induced by NKT cells [52, 53]. This strengthens the hypothesis that high CD1d expression levels on tumor cells correlate with lower metastasis rates [53]. However, most of the tumor immunosurveillance by type I NKT cells is initiated by Th1 cytokines and is mainly dependent on the recruitment and activation of other cytolytic cell populations. In fact, large amounts of IFN-γ and cross-activation of NK cells are necessary for tumor protection upon α-GalCer stimulation. Cytokines such as IL-12 and IL-18 are also necessary to reach optimal IFN-γ levels, consequently leading to tumor immunity [54-56]. Proof that tumor immunosurveillance by type I NKT cells occurs through CD1d became clear when adoptive transfer of liver DN type I NKT cells from WT into CD1d KO mice (lacking all NKT cells) did not confer protection. In Jα18 KO mice (missing type I but retain type II NKT cells) the NKT cell population was able to be recovered and tumor immunity could be rescued upon NKT cell transfer [31, 57]. Nevertheless, in contrast with CD4+ liver type I NKT cells, protection could only be generated using the DN liver type I NKT subset. From these studies it can be concluded that different subsets of NKT cells can have different functions in tumor immunosurveillance [15]. Surface marker expression, anatomical origin as well as different antigens can alter the immunological capacity and function of NKT cells. Type I NKT cells not only increase protective cell responses but can also enhance tumor immunity by modifying the effects of immunosuppressive cells, such as myeloid-derived suppressor cells (MDSCs), suppressive IL-10 producing neutrophils and T regulatory cells [58-61].

### Suppression of tumor immunity

Type II NKT cells possess an immunosuppressive activity in tumor immunology. By counteracting type I NKT cells and negatively influencing other immune cells they are capable to down-regulate tumor immunosurveillance [62, 63]. CD4+ type II NKT cells are producing more IL-13 and IL-4 than type I cells [64]. By the release of Th2 cytokines, type II NKT cells have been shown to suppress autoimmune T cell responses. The original observation was made in a 15-12RM fibrosarcoma model where CD8^+^ cytotoxic T cells were suppressed by CD4^+^ type II NKT cells through production of IL-13 which in turn induced TGF-β, leading to suppression of the antitumor activity [64, 65]. Later on, a similar observation was also reported in several other solid tumor models such as in a CT26 colon carcinoma lung metastasis model, a subcutaneous CT26-L5 colon carcinoma model, an orthothopic K7M2 osteosarcoma model and a renal cell adenocarcinoma liver metastasis model [66]. CD1d KO mice and Jα18 KO mice were compared side-by-side in different tumor models. CD1d KO mice were resistant to tumor growth while Jα18 KO mice behaved similar to wild type mice. This confirms the hypothesis that type II NKT cells present in Jα18 KO were sufficient for suppression of tumor immunosurveillance. Anti-CD4 treatment was able to abrogate the retained suppression, consistent with the original observation that the suppressing cell type has a CD4^+^ phenotype [66]. Furthermore, direct selective stimulation by sulfatide significantly induced growth of CT26 lung metastasis. The effect was retained in Jα18 KO mice but was lacking in CD1d KO mice. This indicated that the effect of sulfatide was only type II NKT cell specific. As a result, it was assumed that type II NKT cells also suppress anti-tumor immune responses in humans in a similar way [62].

Although the immunosuppressive role is often attributed to type II NKT cells, there are a number of exceptions reported in literature where type I NKT cells appear to support immunosuppression [67-69]. Th2 cytokines (IL-13, TGF-β) produced by type I NKT cells conferred immunosuppression, subsequently leading to the inhibition of cytotoxic T cells and NK cell activity. The outcome of type I NKT cell-activation is dependent on different factors such as the antigens, co-stimulatory signals and the cytokine milieu which determine the plasticity of these cells. Immunosuppressive Tregs have been shown to be supported by activated type I NKT cells through IL-2 production, but subsequently suppressed the NKT cells in a cell-cell contact manner [70]. Two studies have reported type I NKT cells capable of directly suppressing tumor immunity in animal models of hematological malignancies. In a RMA/T cell lymphoma model, NKT deficient mice had augmented cytotoxic T cell activity and greater survival rates than WT mice [68]. In a model of Burkitt's-like B cell lymphoma, Jα18 KO mice had significantly fewer splenic tumors than WT or CD1d KO mice. Stimulation of type I NKT cells with α-GalCer did not increase tumor burden, it decreased tumor specific CD8+ T cells [67].

### Cross regulation

It has been demonstrated that type I and type II NKT subsets not only exert positive or negative effects on different cell populations but also cross-regulate each other. CD1d KO mice, deficient in both NKT types, showed strong resistance towards tumor growth in the CT26 colon carcinoma model, whereas Jα18 KO mice, lacking only type I NKT cells, showed higher sensitivity to tumor growth than WT mice. Consequently, this suggests that type I NKT cells may reduce the suppressive effect of type II NKT cells [62]. The *in vivo* activation of type II NKT cells by sulfatide enhanced new tumor formation, and abrogated or reduced the positive clinical effects of α-GalCer when administered together. For example, decreased pro-inflammatory cytokine secretion was observed, thereby indicating that type II NKT cells may also have the ability to suppress type I NKT cell activation. Addition of the type II antigen sulfatide *in vitro* inhibited α-GalCer-induced IFN-γ, IL-2, and IL-4 production [50, 58, 59]. Moreover, Halder RC et al demonstrated by use of the model of concanavalin A-induced hepatitis, that the activation of sulfatide-reactive type II NKT cells and plasmacytoid DCs in the liver contributed to the anergy, or hyporesponsiveness of type I NKT cells [73]. However, it is unclear if this mechanism is similar in a tumor setting. Nevertheless, the finding that immune-protective type I and immune-suppressive type II NKT cells cross-regulate each other establishes an NKT regulatory axis and creates the opportunity to exploit this knowledge in a clinical setting. The success of immunotherapies may depend on which way the balance of the axis is shifted. Enhancing the activity of type I NKT cells while simultaneously blocking type II NKT cells could be a promising strategy for future anti-tumor therapies [74].

## NATURAL KILLER T CELLS: IMPLICATIONS IN MULTIPLE MYELOMA

Multiple Myeloma (MM) is an incurable monoclonal plasma cell malignancy, located primarily in the bone marrow (BM), with an incidence of 5 per 100 000 inhabitants, and affecting approximately 25 000 new patients yearly in the EU [75]. This haematological malignancy is characterized by the secretion of M-proteins in the serum and the urine. Within the BM microenvironment different cell types are undergoing an evolving crosstalk, promoting tumor cell survival and progression. The interwoven stroma provides the supporting framework of the tumor, including extracellular matrix proteins, growth factors, cellular interactions with fibroblasts, macrophages, endothelial cells, bone cells (osteoblasts and osteoclasts) and adipocytes. In turn, MM dysregulates the BM, resulting in osteolytic lesions, anemia, renal failure, and immunosuppression. The overall survival of treated patients below 68 is 8-9 years; the event free survival is 3-4 years [76]. First-line therapy includes high dose corticosteroids (dexamethasone) and other cytolytic agents such as bortezomib, thalidomide and lenalidomide (LEN) or melphalan [77-81]. Depending on the status of the patient this can then be followed by autologous stem cell transplantation (ASCT). Consolidation and maintenance therapy after ASCT are attractive strategies to increase the beneficial effects. However, even after this intensive treatment, in the majority of patients some MM cells remain. This is called “minimal residual disease” (MRD) [81]. Eventually, these cells can grow out, induce relapse and patients ultimately become refractory to all treatment options. Despite the different chemotherapeutic modalities and the many clinical trials to eradicate this disease, MM is still incurable.

**Figure 2:**
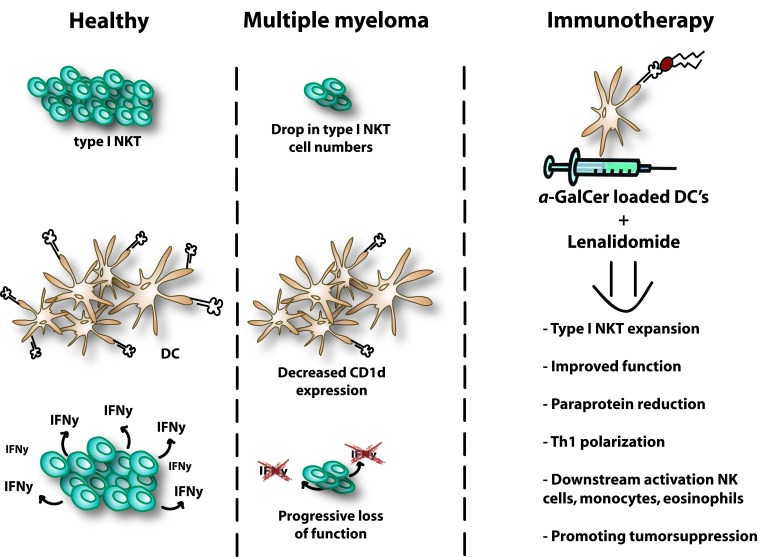
NKT dysregulation in multiple myeloma Type I NKT cells are quantitatively and qualitatively defective in MM which hampers their anti-tumor potential. A dramatic drop of type I NKT cell number can be observed in tumor mouse models and MM patients. CD1d expression levels are down-regulated in advanced MM patients and expression is lost in most of the studied myeloma cell lines. Moreover, advanced stages of MM are associated with a progressive loss of function and their capacity to secrete IFN-γ. LEN has the ability to induce type I NKT expansion in presence of α-GalCer and to stimulate IFN-γ secretion by NKT cells in MM patients. Combination therapy provides downstream activation of NK cells, monocytes and eosinophils and ultimately promotes tumor suppression. A reduction of paraprotein is detected in the serum or urine. NKT, Natural killer T; DC, dendritic cells; NK, natural killer; Th, T helper; α-GalCer, Alpha-Galactosylceramide; IFN, Interferon.

Different research groups provided evidence of type I NKT cells being quantitatively and qualitatively defective in MM, subsequently hampering their anti-tumor effects [82] (Figure 2). The group of Dhodapkar et al. demonstrated that type I NKT cells are still detectable in the blood and tumor bed of MM patients at both early and progressive stages of the disease but they could observe that advanced stages of MM were associated with the progressive loss of the ability of iNKT cells to secrete IFN-γ. The type I NKT dysfunction could be overcome *in vitro* by using dendritic cells (DCs) pulsed with α-GalCer. When MM patients were injected with αGalCer loaded DCs (at monthly interval 2 injections), their type I NKT cell pool expanded 100 fold, with improved function and these effects lasted for several months. Altogether, these results suggest that clinical progression is linked to an acquired but potentially reversible defect in type I NKT cells and supports the hypothesis that iNKT cells help in controlling the malignant growth of the MM cells [82, 83]. Together with other groups they further demonstrated that MM cells are expressing CD1d and are sensitive to lysis induced by type I NKT cells, making them interesting targets for NKT directed therapies [82]. Spanoudakis et al has shown that CD1d was highly expressed on premalignant and early myeloma. With disease progression CD1d expression levels were down-regulated and eventually lost altogether in advanced MM patients and in most of the studied myeloma cell lines, leading to a reduction in survival [84]. Engagement of CD1d by anti-CD1d monoclonal antibodies was able to induce myeloma cell death *in vitro* which was not induced by caspase-activation but was rather associated with overexpression of the pro-apoptotic protein Bax and mitochondrial membrane potential loss [84].

We recently investigated the number, activity and characteristics of type I NKT cells in the syngeneic preclinical 5T33MM murine model, an immunocompetent model which mimics the human disease closely [85]. Consistent with previous observations, our results demonstrated a dramatic drop of type I NKT cell numbers in the liver and spleen at the end stage of the disease. This decline was also detectable in the 5T2MM model, a slower progressing model. The ability of murine type I NKT cells to secrete IFN-γ in response to α-GalCer loaded mature DCs was abrogated at the end stage of the disease due to a decline in NKT number. Treatment with α-GalCer loaded DCs significantly increased the survival of MM diseased mice for 1 week when they were injected on the same day of 5T33MM inoculation [85]. The group of Mattarollo et al. could also demonstrate that a single vaccination of irradiated tumor cells pulsed with α-GalCer was able to inhibit MM development and prolong survival of Vk*MYC mice [86]. Nonetheless, the expression of CD1d in the 5T33MM model was still high at the end stage of MM and lacked the potency to activate type I NKT cells and cause tumor cell lysis after stimulation with α-GalCer. We also found that the 5T33MM cells lacked the necessary co-stimulatory molecules such as CD40, CD80 and CD86 potentially explaining our observations [85]. Hong et al. however demonstrated that a vaccine consisting of αGalCer-loaded MOPC315BM myeloma cells efficiently promoted anti-tumor immunity, slowed down tumor growth, induced established tumor regression and protected (surviving) mice from tumor rechallenge. Strong humoral immune responses, including myeloma-specific antibodies and cellular immune responses, such as myeloma-specific CD8^+^cytotoxic and memory T cells were induced and Treg cells were significantly decreased [87]. It is known that MM correlates with a high vascular index. Targeting angiogenesis is therefore an important therapeutic tool to reduce MM progression. We were able to demonstrate that the conditioned medium of α-GalCer stimulated NKT cells induced a reduction in endothelial cell proliferation, migration and network formation and increased their apoptosis *in vitro*, whereby the JAK-STAT signalling pathway was highly activated. Furthermore, injecting α-GalCer *in vivo* led to a significant reduction in microvessel density [88].

Song et al. succeeded in activating and expanding CD1d-restricted type I NKT cell lines isolated from newly diagnosed and advanced MM patients [89]. The results showed that type I NKT cells could secrete Th1-polarized cytokines in response to α-GalCer loaded DCs or primary MM cells and that they could induce direct cytotoxicity against the primary MM cells. LEN, a derivate of thalidomide and one of the novel drugs used to treat MM, is effective in inducing complete or good partial responses and is able to improve the survival of MM patients [74]. It has among others immunomodulatory properties, although the specific cellular targets and molecular mechanisms responsible for the immunomodulatory actions of LEN have not been fully elucidated yet. Song et al. provided preclinical evidence that a combination of type I NKT immunotherapy with LEN led to an increased Th1 cytokine production and reduced Th2 cytokine levels [89] (Figure 2). The group of Chang et al. observed an even greater effect when LEN was combined with dexamethasone [83]. They further obtained striking results when α-GalCer loaded DCs were injected in 3 MM patients at stage III. Intravenous injection of α-GalCer loaded mature DCs in these patients, who had received chemotherapy and stem cell transplantation, gave a remarkable boost in the expansion of circulating type I NKT cells which sequentially resulted in a reduction of the serum and urine levels of M-protein. A sustained expansion of type I NKT cells, lasting 3 months after vaccination, was observed in one of the patients. An increase of different factors such as IL-12 p40, IP-10 and MIP-1β in the patient serum levels were detected [83]. Confirming previous *in vitro* results, Dhodapkar et al found that LEN had the ability to enhance type I NKT expansion in presence of α-GalCer and to stimulate IFN-γ secretion by NKT cells in both healthy donors and MM patients (Figure 2). The combination therapy provided downstream activation of NK cells, monocytes and eosinophils by upregulating surface receptors such as NKG2D, CD56 and CD16, ultimately promoting tumor suppression [90].

Data on type II NKT cells and MM are scarcely present in literature. However, their increase in the peripheral blood of MM patients was reported by Chang DH et al. Those type II NKT cells appeared to be specific for lyso-PC and had a Th2-skewed profile with high expression levels of IL-13 [40]. Taken together, these data suggest that NKT cells are a particularly attractive subset to target and encourage the rationale for type I NKT cell-mediated immunotherapy in MM.

## NATURAL KILLER T CELLS: CHALLENGES

The use of α-GalCer and other glycolipids to activate type I NKT cells has engendered a lot of preclinical success in mice, leading to multiple clinical trials in humans (Table 1). However, the benefits for patients remains limited since the translation of these preclinical benefits into clinical trials is associated with some challenges [91]. As noted above, the frequency of type I NKT cells is much lower in humans than in mice and numbers are more variable between individuals which possibly can contribute to the heterogeneity in clinical responses [16, 92, 93]. In advanced stages of cancers, like MM, the number and function of type I NKT cells is often reduced. Therefore, the effects of NKT activation may be less amplified in humans than in mice [91]. Moreover, we can also presume that patients that participated in these trials had a much more advanced disease than mice in which α-GalCer had a significant greater therapeutic effect. It is worth to mention that it has been demonstrated in mice that following injection of α-GalCer, type I NKT cells cannot be restimulated for at least two months [94-96]. This means they are sensitive to anergy which is a property that can also explain the lack of success in humans. Being confronted in our research with this problem as well we have the opinion that this is one of the major obstacles which need to be overcome to give a future to an NKT-based immunotherapeutic approach. Specifically, marked increase in programmed death-1 (PD-1) expression after α-GalCer stimulation has been shown to hamper the beneficial and/or long lasting effects of NKT cell-mediated treatment [97]. We also believe that a suboptimal activation due to uncontrolled distribution of α-GalCer remains a big problem. Therefore, it would be of great value to develop new α-GalCer carrier systems (e.g. nanovectors, liposomes and exosomes) to optimize NKT-cell responses and cancer immunotherapy [100, 101]. Also the different (sub)populations, their adaptable reactivity against ligand agonists and the different APCs involved in the antigen presentation add more complexity and can explain the paradox regarding the NKT cell subpopulations. Furthermore, it is possible that the presence of different endogenous self-antigens, leading to auto-reactivity, can activate different pathways in the NKTs that are modulating the NKT cell - cell talk [102]. Finally, our knowledge of the presence of endogenous ligands is still very limited hampering our true understanding of NKT cell biology [27].

**Tabel 1:** Brief overview of α-GalCer–based clinical trials in different cancers

Therapeutic setting	Cancer type	Clinical outcome	Immunological responses	References
**α-GalCer (i.v.)**	Solid tumors	7 out of 24 patients had stable disease	Increase in IL-12, GM-CSF and TNF-α, serum levels	[103]
**α-GalCer-pulsed immature MoDCs (i.v. & i.d.)**	metastatic malignancies	2 patients out of 12 with decreased tumor markers in serum, 1 with tumor necrosis	Expansion NKT cells, activation of T and NK cells, increased IFN-γ levels	[30]
**α-GalCer-pulsed immature MoDCs (i.v)**	Non-small cell lung cancer	5 out of 9 had no change in disease status, 4 patients had disease progression, but 1 case had increase in NKT cells, 2 cases had significant responses	Expansion NKT cells, increase in IFN-γ mRNA levels	[104]
**α-GalCer-pulsed mature MoDCs (i.v)**	Anal cancer Renal cell cancer Multiple myeloma	The patient had stable disease The patient had stable disease The 3 patients had decreased levels of paraprotein in serum or urine	Expansion NKT cells and antigen-specific memory T cells	[83]
***Ex vivo* expanded NKT cells with autologous α-GalCer-pulsed PBMCs (i.v.)**	Non-small cell lung cancer	4 out of 6 patients had a stable disease, 2 patients had disease progression	Expansion NKT cells, elevated IFN-γ cell number	[105]
**α-GalCer-pulsed autologous APCs (via nasal submucosa)**	Head and neck squamous cell carcinoma	1 out of 9 patient had a partial response, 5 patients had a stable disease, 3 patients had disease progression	Expansion NKT cells, elevated IFN-γ cell number in tumor tissue and PBMCs	[106]
**α-GalCer-pulsed APCs (i.v.)**	Non-small cell lung cancer	5 out of 17 patients had a stable disease, the remaining 12 had disease progression	Expansion NKT cells, elevated IFN-γ cell number in tumor tissue and PBMCs	[107]
***In vitro* expanded NKT cells (i.a.) and α-GalCer-pulsed APCs (via nasal submucosa)**	Head and neck squamous cell carcinoma	1 out of 8 patients had disease progression, 3 patients reacted partially, 4 patients had a stable disease	Expansion NKT cells, elevated IFN-γ cell number in tumor tissue and PBMCs	[108]
***In vitro* expanded NKT cells (i.a.) and α-GalCer-pulsed APCs (via nasal submucosa)**	Head and neck squamous cell carcinoma	5 out of 10 patients reacted partially, 5 patients had a stable disease	Expansion NKT cells, elevated IFN-γ cell number in tumor tissue and PBMCs	[109]
**α-GalCer-pulsed mature MoDCs (i.v) and LEN (oral, 10mg/day, 3 28 day cycles)**	Multiple myeloma	3 out of 6 patients with decreased levels of paraprotein in serum or urine	NKT, NK, monocyte and eosinophil activation	[90]

α-GalCer: alpha- galactosylceramide; APCs: antigen-presenting cells; GM-CSF: granulocyte macrophage colonystimulating factor; i.a.: intraarterial; i.d.:intradermal; i.v.: intravenous; MoDCs: monocyte-derived dendritic cells; NKT: natural killer T cells; PBMCs: peripheral blood mononuclear cells; TNF-α: tumor necrosis factor- alpha; LEN: lenalidomide

## References

[1] Godfrey DI, Hammond KJL, Poulton LD, Smyth MJ, Baxter AG (2000). NKT cells: Facts, functions and fallacies. Immunol Today.

[2] Rossjohn J, Pellicci DG, Patel O, Gapin L, Godfrey DI (2012). Recognition of CD1d-restricted antigens by natural killer T cells. Nat Rev Immunol.

[3] Terabe M, Berzofsky J (2008). The Role of NKT Cells in Tumor Immunity. Adv Cancer Res.

[4] Godfrey DI, MacDonald HR, Kronenberg M, Smyth MJ, Van Kaer L (2004). NKT cells: what's in a name?. Nat Rev Immunol.

[5] Matsuda JL, Naidenko O V, Gapin L, Nakayama T, Taniguchi M, Wang CR, Koezuka Y, Kronenberg M (2000). Tracking the response of natural killer T cells to a glycolipid antigen using CD1d tetramers. J Exp Med.

[6] Godfrey DI, Kronenberg M (2004). Going both ways: Immune regulation *via* CD1d-dependent NKT cells. J Clin Invest.

[7] Robertson FC, Berzofsky J, Terabe M (2014). NKT Cell Networks in the Regulation of Tumor Immunity. Front Immunol.

[8] Wu L, Gabriel CL, Parekh V V, Van Kaer L (2009). Invariant natural killer T cells: Innate-like T cells with potent immunomodulatory activities. Tissue Antigens.

[9] Brennan PJ, Brigl M, Brenner MB (2013). Invariant natural killer T cells: an innate activation scheme linked to diverse effector functions. Nat Rev Immunol.

[10] Van Kaer L (2004). Regulation of immune responses by CD1d-restricted natural killer T cells. Immunol Res.

[11] Van Der Vliet HJJ, Molling JW, Von Blomberg BME, Nishi N, Kölgen W, Van Den Eertwegh AJM, Pinedo HM, Giaccone G, Scheper RJ (2004). The immunoregulatory role of CD1d-restricted natural killer T cells in disease. Clin Immunol.

[12] Gumperz JE, Miyake S, Yamamura T, Brenner MB (2002). Functionally distinct subsets of CD1d-restricted natural killer T cells revealed by CD1d tetramer staining. J Exp Med.

[13] Bendelac A, Killeen N, Litteman DR, Schwartz RH (1994). A subset of CD4+ thymocytes selected by MHC class I molecules. Science.

[14] Takahashi T, Chiba S, Nieda M, Azuma T, Ishihara S, Shibata Y, Juji T, Hirai H (2002). Cutting Edge: Analysis of Human V 24+CD8+ NK T Cells Activated by -Galactosylceramide-Pulsed Monocyte-Derived Dendritic Cells. J Immunol.

[15] Crowe NY, Coquet JM, Berzins SP, Kyparissoudis K, Keating R, Pellicci DG, Hayakawa Y, Godfrey DI, Smyth MJ (2005). Differential antitumor immunity mediated by NKT cell subsets *in vivo*. J Exp Med.

[16] Bendelac A, Savage PB, Teyton L (2007). The biology of NKT cells. Annu Rev Immunol.

[17] Lynch L, O'shea D, Winter DC, Geoghegan J, Doherty DG, O'Farrelly C (2009). Invariant NKT cells and CD1d+ cells amass in human omentum and are depleted in patients with cancer and obesity. Eur J Immunol.

[18] Kobayashi E, Motoki K, Uchida T, Fukushima H, Koezuka Y (1995). KRN7000, a novel immunomodulator, and its antitumor activities. Oncol Res.

[19] Aspeslagh S, Li Y, Yu ED, Pauwels N, Trappeniers M, Girardi E, Decruy T, Van Beneden K, Venken K, Drennan M, Leybaert L, Wang J, Franck RW (2011). Galactose-modified iNKT cell agonists stabilized by an induced fit of CD1d prevent tumour metastasis. EMBO J.

[20] Kinjo Y, Wu D, Kim G, Xing G-W, Poles M a, Ho DD, Tsuji M, Kawahara K, Wong C-H, Kronenberg M (2005). Recognition of bacterial glycosphingolipids by natural killer T cells. Nature.

[21] Kinjo Y, Illarionov P, Vela J, Pei B, Girardi E, Li X, Li Y, Imamura M, Kaneko Y, Okawara a, Miyazaki Y, Gómez-Velasco a, Rogers P (2011). Invariant natural killer T cells recognize glycolipids from pathogenic Gram-positive bacteria.

[22] Mattner J, Debord KL, Ismail N, Goff RD, Cantu C, Zhou D, Saint-Mezard P, Wang V, Gao Y, Yin N, Hoebe K, Schneewind O, Walker D (2005). Exogenous and endogenous glycolipid antigens activate NKT cells during microbial infections. Nature.

[23] Chang YJ, Kim HY, Albacker L a, Lee HH, Baumgarth N, Akira S, Savage PB, Endo S, Yamamura T, Maaskant J, Kitano N, Singh A, Bhatt A (2011). Influenza infection in suckling mice expands an NKT cell subset that protects against airway hyperreactivity. J Clin Invest.

[24] Ito Y, Vela JL, Matsumura F, Hoshino H, Tyznik A, Lee H, Girardi E, Zajonc DM, Liddington R, Kobayashi M, Bao X, Bugaytsova J, Borén T (2013). Helicobacter pylori cholesteryl α-glucosides contribute to its pathogenicity and immune response by natural killer T cells. PLoS One.

[25] Anderson B. L, Teyton L, Bendelac A, Savage B. P. (2013). Stimulation of Natural Killer T cells by Glycolipids. Molecules.

[26] Birkholz a. M, Girardi E, Wingender G, Khurana a., Wang J, Zhao M, Zahner S, Illarionov P a., Wen X, Li M, Yuan W, Porcelli S a., Besra GS (2015). A Novel Glycolipid Antigen for NKT Cells That Preferentially Induces IFN- Production. J Immunol.

[27] Kain L, Webb B, Anderson BL, Deng S, Holt M, Costanzo A, Zhao M, Self K, Teyton A, Everett C, Kronenberg M, Zajonc DM, Bendelac A (2014). The Identification of the Endogenous Ligands of Natural Killer T Cells Reveals the Presence of Mammalian α-Linked Glycosylceramides. Immunity.

[28] Galili U, Shohet SB, Kobrin E, Stults CL, Macher B a (1988). Man, apes, and Old World monkeys differ from other mammals in the expression of alpha-galactosyl epitopes on nucleated cells. J Biol Chem.

[29] Kawano T, Cui J, Koezuka Y, Toura I, Kaneko Y, Sato H, Kondo E, Harada M, Koseki H, Nakayama T, Tanaka Y, Taniguchi M (1998). Natural killer-like nonspecific tumor cell lysis mediated by specific ligand-activated Valpha14 NKT cells. Proc Natl Acad Sci U S A.

[30] Nieda M, Okai M, Tazbirkova A, Lin H, Yamaura A, Ide K, Abraham R, Juji T, Macfarlane DJ, Nicol AJ (2004). Therapeutic activation of Valpha24+Vbeta11+NKT cells in human subjects results in highly coordinated secondary activation of acquired and innate immunity. Cell.

[31] Smyth MJ, Thia KY, Street SE, Cretney E, Trapani J a, Taniguchi M, Kawano T, Pelikan SB, Crowe NY, Godfrey DI (2000). Differential tumor surveillance by natural killer (NK) and NKT cells. J Exp Med.

[32] Van Kaer L, Parekh V V, Wu L (2013). Invariant natural killer T cells as sensors and managers of inflammation. Trends Immunol.

[33] Brigl M, Bry L, Kent SC, Gumperz JE, Brenner MB (2003). Mechanism of CD1d-restricted natural killer T cell activation during microbial infection. Nat Immunol.

[34] Brigl M, Tatituri RV V, Watts GFM, Bhowruth V, Leadbetter E a, Barton N, Cohen NR, Hsu F-F, Besra GS, Brenner MB (2011). Innate and cytokine-driven signals, rather than microbial antigens, dominate in natural killer T cell activation during microbial infection. J Exp Med.

[35] Arrenberg P, Halder R, Dai Y, Maricic I, Kumar V (2010). Oligoclonality and innate-like features in the TCR repertoire of type II NKT cells reactive to a beta-linked self-glycolipid. Proc Natl Acad Sci U S A.

[36] Arrenberg P, Halder R, Kumar V (2009). Cross-regulation between distinct natural killer T cell subsets influences immune response to self and foreign antigens. J Cell Physiol.

[37] Roy KC, Maricic I, Khurana A, Smith TRF, Halder RC, Kumar V (2008). Involvement of secretory and endosomal compartments in presentation of an exogenous self-glycolipid to type II NKT cells. J Immunol.

[38] Jahng A, Maricic I, Aguilera C, Cardell S, Halder RC, Kumar V (2004). Prevention of autoimmunity by targeting a distinct, noninvariant CD1d-reactive T cell population reactive to sulfatide. J Exp Med.

[39] Rhost S, Löfbom L, Rynmark BM, Pei B, Månsson JE, Teneberg S, Blomqvist M, Cardell SL (2012). Identification of novel glycolipid ligands activating a sulfatide-reactive, CD1d-restricted, type II natural killer T lymphocyte. Eur J Immunol.

[40] Chang DH, Deng H, Matthews P, Krasovsky J, Ragupathi G, Spisek R, Mazumder A, Vesole DH, Jagannath S, Dhodapkar M V (2008). Inflammation-associated lysophospholipids as ligands for CD1d-restricted T cells in human cancer. Blood.

[41] Godfrey DI, Pellicci DG, Patel O, Kjer-Nielsen L, McCluskey J, Rossjohn J (2010). Antigen recognition by CD1d-restricted NKT T cell receptors. Semin Immunol.

[42] Van Rhijn I, Young DC, Im JS, Levery SB, Illarionov P a, Besra GS, Porcelli S a, Gumperz J, Cheng T-Y, Moody DB (2004). CD1d-restricted T cell activation by nonlipidic small molecules. Proc Natl Acad Sci U S A.

[43] Tilloy F, Treiner E, Park SH, Garcia C, Lemonnier F, de la Salle H, Bendelac a, Bonneville M, Lantz O (1999). An invariant T cell receptor alpha chain defines a novel TAP-independent major histocompatibility complex class Ib-restricted alpha/beta T cell subpopulation in mammals. J Exp Med.

[44] Patel O, Kjer-Nielsen L, Le Nours J, Eckle SB, Birkinshaw R, Beddoe T, Corbett AJ, Liu L, Miles JJ, Meehan B, Reantragoon R, Sandoval-Romero ML, Sullivan LC, Brooks AG (2013). Recognition of vitamin B metabolites by mucosal-associated invariant T cells. Nat Commun..

[45] Kjer-Nielsen L, Patel O, Corbett AJ, Le Nours J, Meehan B, Liu L, Bhati M, Chen Z, Kostenko L, Reantragoon R, Williamson NA, Purcell AW, Dudek NL, McConville MJ (2012). MR1 presents microbial vitamin B metabolites to MAIT cells. Nature.

[46] McWilliam HE, Birkinshaw RW, Villadangos J a, McCluskey J, Rossjohn J (2015). MR1 presentation of vitamin B-based metabolite ligands. Curr Opin Immunol.

[47] Treiner E, Lantz O (2006). CD1d- and MR1-restricted invariant T cells: of mice and men. Curr Opin Immunol.

[48] Reantragoon R, Corbett a J, Sakala IG, Gherardin N a., Furness JB, Chen Z, Eckle SBG, Uldrich a. P, Birkinshaw RW, Patel O, Kostenko L, Meehan B, Kedzierska K (2013). Antigen-loaded MR1 tetramers define T cell receptor heterogeneity in mucosal-associated invariant T cells. J Exp Med.

[49] Carding SR, Egan PJ (2002). Gammadelta T cells: functional plasticity and heterogeneity. Nat Rev Immunol.

[50] Lafont V, Sanchez F, Laprevotte E, Michaud H-A, Gros L, Eliaou J-F, Bonnefoy N (2014). Plasticity of gamma delta T Cells: Impact on the Anti-Tumor Response. Front Immunol.

[51] Coquet JM, Kyparissoudis K, Pellicci DG, Besra G, Berzins SP, Smyth MJ, Godfrey DI (2007). IL-21 is produced by NKT cells and modulates NKT cell activation and cytokine production. J Immunol.

[52] Fallarini S, Paoletti T, Orsi Battaglini N, Lombardi G (2012). Invariant NKT cells increase drug-induced osteosarcoma cell death. Br J Pharmacol.

[53] Hix LM, Shi YH, Brutkiewicz RR, Stein PL, Wang CR, Zhang M (2011). CD1d-expressing breast cancer cells modulate NKT cell-mediated antitumor immunity in a murine model of breast cancer metastasis. PLoS One.

[54] Street SE a, Cretney E, Smyth MJ (2001). Perforin and interferon-γ activities independently control tumor initiation, growth, and metastasis. Blood.

[55] Gonzalez-Aseguinolaza G, Van Kaer L, Bergmann CC, Wilson JM, Schmieg J, Kronenberg M, Nakayama T, Taniguchi M, Koezuka Y, Tsuji M (2002). Natural killer T cell ligand alpha-galactosylceramide enhances protective immunity induced by malaria vaccines. J Exp Med.

[56] Smyth MJ, Crowe NY, Pellicci DG, Kyparissoudis K, Kelly JM, Takeda K, Yagita H, Godfrey DI (2002). Sequential production of interferon-gamma by NK1. 1(+) T cells and natural killer cells is essential for the antimetastatic effect of alpha-galactosylceramide. Blood.

[57] Crowe NY, Smyth MJ, Godfrey DI (2002). A critical role for natural killer T cells in immunosurveillance of methylcholanthrene-induced sarcomas. J Exp Med.

[58] Santo C, De Salio M, Masri SH, Lee LY, Dong T, Speak AO, Porubsky S, Booth S, Veerapen N, Besra GS, Gröne H, Platt FM, Zambon M (2008). Invariant NKT cells reduce the immunosuppressive activity of influenza A virus - induced myeloid-derived suppressor cells in mice and humans. Heal (San Fr.).

[59] De Santo C, Arscott R, Booth S, Karydis I, Jones M, Asher R, Salio M, Middleton M, Cerundolo V (2011). Invariant NKT cells modulate the suppressive activity of Serum Amyloid A-differentiated IL-10-secreting neutrophils. Nat Immunol.

[60] Venken K, Seeuws S, Zabeau L, Jacques P, Decruy T, Coudenys J, Verheugen E, Windels F, Catteeuw D, Drennan M, Van Calenbergh S, Lambrecht BN, Yoshimura A, Tavernier J ED (2014). A bidirectional crosstalk between iNKT cells and adipocytes mediated by leptin modulates susceptibility for T cell mediated hepatitis. J Hepatol.

[61] Venken K, Decruy T, Aspeslagh S, Van S, Lambrecht BN, Elewaut D (2015). Bacterial CD1d − Restricted Glycolipids Induce IL-10 Production by Human Regulatory T Cells upon Cross-Talk with Invariant NKT Cells.

[62] Ambrosino E, Terabe M, Halder RC, Peng J, Takaku S, Miyake S, Yamamura T, Kumar V, Berzofsky J a (2007). Cross-regulation between type I and type II NKT cells in regulating tumor immunity: a new immunoregulatory axis. J Immunol.

[63] Renukaradhya GJ, Khan M a, Vieira M, Du W, Gervay-hague J, Brutkiewicz RR (2008). Type I NKT cells protect ( and type II NKT cells suppress ) the host ' s innate antitumor immune response to a B-cell lymphoma. Hematology.

[64] Terabe M, Matsui S, Noben-Trauth N, Chen H, Watson C, Donaldson DD, Carbone DP, Paul WE, Berzofsky J a (2000). NKT cell-mediated repression of tumor immunosurveillance by IL-13 and the IL-4R-STAT6 pathway. Nat Immunol.

[65] Terabe M, Matsui S, Park J-M, Mamura M, Noben-Trauth N, Donaldson DD, Chen W, Wahl SM, Ledbetter S, Pratt B, Letterio JJ, Paul WE, Berzofsky J a (2003). Transforming growth factor-beta production and myeloid cells are an effector mechanism through which CD1d-restricted T cells block cytotoxic T lymphocyte-mediated tumor immunosurveillance: abrogation prevents tumor recurrence. J Exp Med.

[66] Terabe M, Swann J, Ambrosino E, Sinha P, Takaku S, Hayakawa Y, Godfrey DI, Ostrand-Rosenberg S, Smyth MJ, Berzofsky J a (2005). A nonclassical non-Valpha14Jalpha18 CD1d-restricted (type II) NKT cell is sufficient for down-regulation of tumor immunosurveillance. J Exp Med.

[67] Bjordahl RL, Gapin L, Marrack P, Refaeli Y (2012). INKT cells suppress the CD8+ T cell response to a murine burkitt's-like b cell lymphoma. PLoS One.

[68] Renukaradhya GJ, Sriram V, Du W, Gervay-Hague J, Van Kaer L, Brutkiewicz RR (2006). Inhibition of antitumor immunity by invariant natural killer T cells in a T-cell lymphoma model *in vivo*. Int J Cancer.

[69] Yang W, Li H, Mayhew E, Mellon J, Chen PW, Niederkorn JY (2011). NKT cell exacerbation of liver metastases arising from melanomas transplanted into either the eyes or spleens of mice. Investig Ophthalmol Vis Sci.

[70] Jiang S, Game DS, Davies D, Lombardi G, Lechler RI (2005). Activated CD1d-restricted natural killer T cells secrete IL-2: Innate help for CD4+CD25+ regulatory T cells?. Eur J Immunol.

[71] Kanamori M, Tasumi Y, Iyoda T, Ushida M, Inaba K (2012). Sulfatide inhibits alpha-galactosylceramide presentation by dendritic cells. Int Immunol.

[72] Berzofsky J a, Terabe M (2009). The contrasting roles of NKT cells in tumor immunity. Curr Mol Med.

[73] Halder RC, Aguilera C, Maricic I, Kumar V (2007). Type II NKT cell-mediated anergy induction in type I NKT cells prevents inflammatory liver disease. J Clin Invest.

[74] Terabe M, Berzofsky J a (2014). The immunoregulatory role of type I and type II NKT cells in cancer and other diseases. Cancer Immunol Immunother.

[75] Sirohi B, Powles R (2004). Multiple myeloma. Lancet.

[76] Barlogie B, Anaissie E, Van Rhee F, Shaughnessy JD, Szymonifka J, Hoering A, Petty N, Crowley J (2010). Reiterative survival analyses of total therapy 2 for multiple myeloma elucidate follow-up time dependency of prognostic variables and treatment arms. J Clin Oncol.

[77] Davies FE, Raje N, Hideshima T, Al E (2001). Thalidomide and immunomodulatory derivatives augment natural killer cell cytotoicity in multiple myeloma. Blood.

[78] Gorgun G, Calabrese E, Soydan E, Hideshima T, Perrone G, Bandi M, Cirstea D, Santo L, Hu Y, Tai Y-T, Nahar S, Mimura N, Fabre C (2010). Immunomodulatory effects of lenalidomide and pomalidomide on interaction of tumor and bone marrow accessory cells in multiple myeloma. Blood.

[79] Perez LE, Parquet N, Meads M, Anasetti C, Dalton W (2010). Bortezomib restores stroma-mediated APO2L/TRAIL apoptosis resistance in multiple myeloma. Eur J Haematol.

[80] Li Shirong, Gill Navkiranjit LS (2010). Recent advances of IMiDs in cancer therapy. Curr Opin Immunol.

[81] Ludwig H, Sonneveld P, Davies F, Bladé J, Boccadoro M, Cavo M, Morgan G, de la Rubia J, Delforge M, Dimopoulos M, Einsele H, Facon T, Goldschmidt H (2014). European Perspective on Multiple Myeloma Treatment Strategies in 2014. Oncologist.

[82] Dhodapkar M V, Geller MD, Chang DH, Shimizu K, Fujii S-I, Dhodapkar KM, Krasovsky J (2003). A reversible defect in natural killer T cell function characterizes the progression of premalignant to malignant multiple myeloma. J Exp Med.

[83] Chang DH, Osman K, Connolly J, Kukreja A, Krasovsky J, Pack M, Hutchinson A, Geller M, Liu N, Annable R, Shay J, Kirchhoff K, Nishi N (2005). Sustained expansion of NKT cells and antigen-specific T cells after injection of alpha-galactosyl-ceramide loaded mature dendritic cells in cancer patients. J Exp Med.

[84] Spanoudakis E, Hu M, Naresh K, Terpos E, Melo V, Reid A, Kotsianidis L, Abdalla S, Rahemtulla A, Karadimitris A (2009). Regulation of multiple myeloma survival and progression by CD1d. Blood.

[85] Nur H, Fostier K, Aspeslagh S, Renmans W, Bertrand E, Leleu X, Favreau M, Breckpot K, Schots R, de Waele M, van Valckenborgh E, de Bruyne E, Facon T (2013). Preclinical Evaluation of Invariant Natural Killer T Cells in the 5T33 Multiple Myeloma Model. PLoS One.

[86] Mattarollo SR, West AC, Steegh K, Duret H, Paget C, Martin B, Matthews GM, Shortt J, Chesi M, Bergsagel PL, Bots M, Zuber J, Lowe SW (2012). NKT cell adjuvant-based tumor vaccine for treatment of myc oncogene-driven mouse B-cell lymphoma. Blood.

[87] Hong S, Lee H, Jung K, Lee SM, Lee SJ, Jun HJ, Kim Y, Song H, Bogen B, Choi I (2013). Tumor cells loaded with α-galactosylceramide promote therapeutic NKT-dependent anti-tumor immunity in multiple myeloma. Immunol Lett.

[88] Nur H, Rao L, Frassanito MA, De Raeve H, Ribatti D, Mfopou JK, Van Valckenborgh E, De Bruyne E, Vacca A, Vanderkerken K, Menu E (2014). Stimulation of invariant natural killer T cells by α-Galactosylceramide activates the JAK-STAT pathway in endothelial cells and reduces angiogenesis in the 5T33 multiple myeloma model. Br J Haematol.

[89] Song W, Van Der Vliet HJJ, Tai YT, Prabhala R, Wang R, Podar K, Catley L, Shammas M a, Anderson KC, Balk SP, Exley M a., Munshi NC (2008). Generation of antitumor invariant natural killer T cell lines in multiple myeloma and promotion of their functions *via* lenalidomide: A strategy for immunotherapy. Clin Cancer Res.

[90] Richter J, Neparidze N, Zhang L, Nair S, Monesmith T, Sundaram R, Miesowicz F, Dhodapkar KM, Dhodapkar M V (2013). Clinical regressions and broad immune activation following combination therapy targeting human NKT cells in myeloma. Blood.

[91] Dhodapkar M V, Richter J (2011). Harnessing natural killer T (NKT) cells in human myeloma: Progress and challenges. Clin Immunol.

[92] Kronenberg M (2005). Toward an understanding of NKT cell biology: progress and paradoxes. Annu Rev Immunol.

[93] Lee PT, Putnam A, Benlagha K, Teyton L, Gottlieb P a, Bendelac A (2002). Testing the NKT cell hypothesis of human IDDM pathogenesis. J Clin Invest.

[94] Fujii S, Shimizu K, Kronenberg M, Steinman RM (2002). Prolonged IFN-gamma-producing NKT response induced with alpha-galactosylceramide-loaded DCs. Nat Immunol.

[95] Parekh V V, Lalani S, Kim S, Halder R, Azuma M, Yagita H, Kumar V, Wu L, Kaer L Van (2009). PD-1/PD-L blockade prevents anergy induction and enhances the anti-tumor activities of glycolipid-activated invariant NKT cells. J Immunol.

[96] Chang W-S, Kim J-Y, Kim Y-J, Kim Y-S, Lee J-M, Azuma M, Yagita H, Kang C-Y (2008). Cutting edge: Programmed death-1/programmed death ligand 1 interaction regulates the induction and maintenance of invariant NKT cell anergy. J Immunol.

[97] Parekh V V, Wilson MT, Olivares-Villagómez D, Singh AK, Wu L, Wang CR, Joyce S, Van Kaer L (2005). Glycolipid antigen induces long-term natural killer T cell anergy in mice. J Clin Invest.

[98] Kadri N, Korpos E, Gupta S, Briet C, Löfbom L, Yagita H, Lehuen A, Boitard C, Holmberg D, Sorokin L, Cardell SL (2012). CD4(+) type II NKT cells mediate ICOS and programmed death-1-dependent regulation of type 1 diabetes. J Immunol.

[99] Swaika A, Hammond W a, Joseph RW (2015). Current state of anti-PD-L1 and anti-PD-1 agents in cancer therapy. Mol Immunol.

[100] Faveeuw C, Trottein F (2014). Optimization of natural killer T cell-mediated immunotherapy in cancer using cell-based and nanovector vaccines. Cancer Res.

[101] Gehrmann U, Hiltbrunner S, Georgoudaki AM, Karlsson MC, Näslund TI, Gabrielsson S (2013). Synergistic induction of adaptive antitumor immunity by codelivery of antigen with α-galactosylceramide on exosomes. Cancer Res.

[102] Wang X, Chen X, Rodenkirch L, Simonson W, Wernimont S, M R, Veerapen N, Gibson D, Howell AR, Besra GS, Painter GF, Huttenlocher A, Gumperz JE (2008). Natural killer T-cell autoreactivity leads to a specialized activation state. Blood.

[103] Giaccone G, Punt CJ a, Ando Y, Ruijter R, Nishi N, Peters M, von Blomberg BME, Scheper RJ, van der Vliet HJJ, van den Eertwegh AJM, Roelvink M, Beijnen J, Zwierzina H (2002). A phase I study of the natural killer T-cell ligand alpha-galactosylceramide (KRN7000) in patients with solid tumors. Clin Cancer Res.

[104] Ishikawa A, Motohashi S, Ishikawa E, Fuchida H, Higashino K, Otsuji M, Iizasa T, Nakayama T, Taniguchi M, Fujisawa T (2005). A phase I study of alpha-galactosylceramide (KRN7000)-pulsed dendritic cells in patients with advanced and recurrent non-small cell lung cancer. Clin Cancer Res.

[105] Motohashi S, Ishikawa A, Ishikawa E, Otsuji M, Iizasa T, Hanaoka H, Shimizu N, Horiguchi S, Okamoto Y, Fujii S, Taniguchi M, Fujisawa T, Nakayama T (2006). A phase I study of *in vitro* expanded natural killer T cells in patients with advanced and recurrent non-small cell lung cancer. Clin Cancer Res.

[106] Uchida T, Horiguchi S, Tanaka Y, Yamamoto H, Kunii N, Motohashi S, Taniguchi M, Nakayama T, Okamoto Y (2008). Phase I study of α-galactosylceramide-pulsed antigen presenting cells administration to the nasal submucosa in unresectable or recurrent head and neck cancer. Cancer Immunol Immunother.

[107] Motohashi S, Nagato K, Kunii N, Yamamoto H, Yamasaki K, Okita K, Hanaoka H, Shimizu N, Suzuki M, Yoshino I, Taniguchi M, Fujisawa T, Nakayama T (2009). A phase I-II study of alpha-galactosylceramide-pulsed IL-2/GM-CSF-cultured peripheral blood mononuclear cells in patients with advanced and recurrent non-small cell lung cancer. J Immunol.

[108] Kunii N, Horiguchi S, Motohashi S, Yamamoto H, Ueno N, Yamamoto S, Sakurai D, Taniguchi M, Nakayama T, Okamoto Y (2009). Combination therapy of *in vitro*-expanded natural killer T cells and alpha-galactosylceramide-pulsed antigen-presenting cells in patients with recurrent head and neck carcinoma. Cancer Sci.

[109] Yamasaki K, Horiguchi S, Kurosaki M, Kunii N, Nagato K, Hanaoka H, Shimizu N, Ueno N, Yamamoto S, Taniguchi M, Motohashi S, Nakayama T, Okamoto Y (2011). Induction of NKT cell-specific immune responses in cancer tissues after NKT cell-targeted adoptive immunotherapy. Clin Immunol.

